# Synthesis and Performance Evaluation of Novel Bentonite-Supported Nanoscale Zero Valent Iron for Remediation of Arsenic Contaminated Water and Soil

**DOI:** 10.3390/molecules28052168

**Published:** 2023-02-25

**Authors:** Md Basit Raza, Siba Prasad Datta, Debasis Golui, Mandira Barman, Tapas Kumar Das, Rabi Narayan Sahoo, Devi Upadhyay, Mohammad Mahmudur Rahman, Biswaranjan Behera, A Naveenkumar

**Affiliations:** 1 Division of Soil Science and Agricultural Chemistry, ICAR-Indian Agricultural Research Institute, New Delhi 110012, India; 2 ICAR-Indian Institute of Soil and Water Conservation, RC Koraput, Odisha 763002, India; 3 Department of Civil, Construction and Environmental Engineering, North Dakota State University, Fargo, ND 58102, USA; 4 Division of Agronomy, ICAR-Indian Agricultural Research Institute, New Delhi 110012, India; 5 Division of Agricultural Physics, ICAR-Indian Agricultural Research Institute, New Delhi 110012, India; 6 Global Centre for Environmental Remediation (GCER), College of Engineering, Science and Environment, The University of Newcastle, Callaghan, NSW 2308, Australia; 7 Department of General Educational Development, Faculty of Science & Information Technology, Daffodil International University, Ashulia, Savar, Dhaka 1207, Bangladesh; 8 ICAR-Indian Institute of Water Management, Bhubaneswar 751023, India

**Keywords:** adsorption isotherm, ageing, drinking water, error analysis, kinetics, super-adsorbent

## Abstract

Groundwater arsenic (As) pollution is a naturally occurring phenomenon posing serious threats to human health. To mitigate this issue, we synthesized a novel bentonite-based engineered nano zero-valent iron (nZVI-Bento) material to remove As from contaminated soil and water. Sorption isotherm and kinetics models were employed to understand the mechanisms governing As removal. Experimental and model predicted values of adsorption capacity (*q_e_* or *q_t_*) were compared to evaluate the adequacy of the models, substantiated by error function analysis, and the best-fit model was selected based on corrected Akaike Information Criterion (AICc). The non-linear regression fitting of both adsorption isotherm and kinetic models revealed lower values of error and lower AICc values than the linear regression models. The pseudo-second-order (non-linear) fit was the best fit among kinetic models with the lowest AICc values, at 57.5 (nZVI-Bare) and 71.9 (nZVI-Bento), while the Freundlich equation was the best fit among the isotherm models, showing the lowest AICc values, at 105.5 (nZVI-Bare) and 105.1 (nZVI-Bento). The adsorption maxima (*q_max_*) predicted by the non-linear Langmuir adsorption isotherm were 354.3 and 198.5 mg g^−1^ for nZVI-Bare and nZVI-Bento, respectively. The nZVI-Bento successfully reduced As in water (initial As concentration = 5 mg L^−1^; adsorbent dose = 0.5 g L^−1^) to below permissible limits for drinking water (10 µg L^−1^). The nZVI-Bento @ 1% (*w*/*w*) could stabilize As in soils by increasing the amorphous Fe bound fraction and significantly diminish the non-specific and specifically bound fraction of As in soil. Considering the enhanced stability of the novel nZVI-Bento (upto 60 days) as compared to the unmodified product, it is envisaged that the synthesized product could be effectively used for removing As from water to make it safe for human consumption.

## 1. Introduction

Arsenic (As), an element in Group 15 of the periodic table, is deemed to be a systemic poison, which upon sustained exposure leads to serious health conditions and even premature death. Arsenic’s notoriety as a deadly element has earned it the moniker of “king of poison”, so unsurprisingly, it is ranked first on the National Priority List (NPL) of hazardous substances prepared by the Agency for Toxic Substances and Disease Registry (ATSDR) and United States Environmental Protection Agency (US EPA) [1]. Because of its variable oxidation state (−3 to +5), it can form covalent bonds with a wide range of elements. Although it is naturally found mostly in combination with oxygen and sulphur, forming various primary and secondary minerals [2], it also exists in organic forms that are less abundant in the environment and relatively less toxic than the inorganic forms [3]. The toxicity of As varies greatly according to its chemical form: inorganic forms (As(III) and As(V)) are the most toxic, followed by monomethylarsonic acid (MMA) and dimethylarsinic acid (DMA) [4]. Drinking water and contaminated soils are the major pathways for entering the food chain. Groundwater pollution of As is presently affecting the lives of around half a billion people worldwide [5]. In India, 20 states and four union territories are affected by the As contamination of groundwater, thus posing a great risk to the health of more than 50 million people [5,6]. The highest As concentration found in groundwater in India is 3700 µg L^−1^, discovered in tube well water samples acquired from Ramnagar village in South 24 Parganas district, West Bengal. This concentration was 370 times higher than the permissible limit of As in drinking water (10 µg L^−1^) [7,8]. Continued ingestion of As-contaminated food and water potentially impairs cellular metabolism, causing detrimental health effects including arsenicosis, blackfoot disease, cancer, and cardiovascular and pulmonary conditions [9].

Various modern conventional methods including reverse osmosis, chemical precipitation, ion exchange, bio-remediation, membrane filtration, electrochemical oxidation, and electrolytic precipitation are capable of removing toxic pollutant elements from water [10]. However, most of these methods are neither cost-effective nor eco-friendly. In contrast, sorption is regarded as a relatively cheaper and environmentally safe technique for the remediation of heavy metal/metalloid (metal(loid)) polluted water [11]. Iron (Fe)-based products, specifically zero-valent iron (ZVI) have been successfully used to remove various inorganic pollutants [12,13] and organic pollutants [14,15,16] from soil and/or water. These products have received significant attention due to their high sorption properties [17]. However, their application is restricted to laboratory conditions due to their poor structural stability, which causes agglomeration, high rates of corrosion, and reduced reactivity, thus making these products unfit for use in pristine form.

Studies focusing on surface modification of ZVIs have been carried out to enhance their properties and longevity. One of the most widely followed methods of synthesizing modified or engineered ZVI is by providing a support or base that can reduce the pace of redox reactions and agglomeration. Clays (aluminosilicates) are among the most effective adsorbents and are an ideal support for nano zero-valent iron (nZVI) because of their inexpensive nature, high adsorption capacity, and wide availability. Various types of clays such as Fuller’s earth [18], zeolite [19], kaolinite [20], and montmorillonite [21] have been successfully employed as base materials for modifying ZVI to decontaminate As-polluted water. Because of the poor adsorption capacity of these sorbents, often a higher dose is necessary for remediation. Unlike other clays, bentonite is a layered phyllosilicate that acts as a natural adsorbent; it is often used in water purification, effluent treatment, and removal of various organic and inorganic pollutants [22,23]. It has been also used for modifying nZVI to enhance the overall efficiency of nano-sorbents to remove both organic and inorganic pollutants. However, such amendment has not been used for the remediation of As from soil and water. Further, the detailed mechanism of As removal by these engineered products needs further investigation so as to employ it for remediation purposes.

Arsenic can be introduced into the environment naturally (geogenic) by dissolution of As-bearing minerals or by human activities such as mining, fossil fuel combustion, indiscriminate use of agrochemicals, etc. [24]. Over-exploitation of groundwater for agricultural production has been identified as the primary cause for the increase in As in top soil to levels above 10 mg kg^−1^ (world average) in some areas of West Bengal, India [25,26]. Methods for arresting transfer of As from soil to plants mostly rely on modifying soil chemical environments, mainly by using iron (Fe), silicon (Si), phosphorus (P), selenium (Se), and carbon (C)-based amendments [27,28]. The available fraction of As in soil is of primary importance during As remediation, because it has a direct influence on its subsequent bioavailability to plant roots [17]. In this regard, the application of nZVI to remediate As contaminated soils could be a promising option due to its fast reactivity, in-situ application, and long-term stabilizing effect on the immobilized As fraction in soil. The distribution of As in association with various soil constituents and the affinity with which it is held decides the extent of availability of As, which can be studied by conducting a sequential extraction procedure (SEP). There is a dearth of studies on the application of nZVI for As remediation in soil and its influence on different As fractions in soil.

Therefore, the present work was carried out to synthesize a novel nano zero-valent iron material with higher stability and high adsorption capacity. An ageing study and X-ray diffraction analysis, along with sorption isotherm and kinetic analyses, were carried out to understand the mechanism behind As removal. The adequacy of the models and selection of the best-fit model were assessed mathematically by validation using various error functions. This work also aimed to standardize the application dose of the novel product on water and soil, and study its effect on the distribution of As within various fractions in soil.

## 2. Results and Discussion

### 2.1. Product Characterization: Morphology and Chemical Properties

#### 2.1.1. Morphological Features

**Figure 1:**
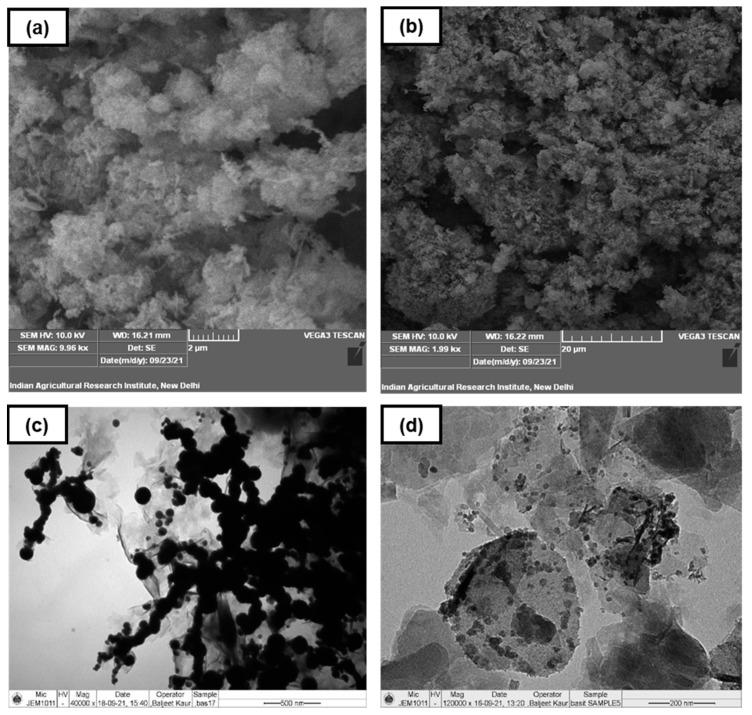
Images showing the characterization of ZVI products using scanning and transmission electron microscopy (SEM and TEM): (**a**,**b**) SEM images of nZVI-Bare and nZVI-Bento, respectively; (**c**,**d**) TEM images of nZVI-Bare and nZVI-Bento, respectively.

Surface characterization of the synthesized products was conducted by scanning electron microscopy (SEM). Because of the higher depth of field (DOF), the SEM micrographs displayed detailed three-dimensional features of the product surface. The surface of bare nano zero-valent iron (nZVI-Bare) was more homogenous and endowed with finer pores compared to bentonite-supported nano zero-valent iron (nZVI-Bento) (Figure 1a,b). The microstructure and morphology at nanoscale could be visualized using transmission electron microscopy (TEM) [29]. The TEM micrographs of the nZVI-Bento clearly exhibited loading of Fe^0^ on the clay surface (Figure 1d). The Fe^0^ particles in nZVI-Bento appeared to be more dispersed when compared to nZVI-Bare.

The images of nZVI-Bare revealed a chain of globular-shaped particles with scattered aggregation (Figure 1c). This might be explained by (i) the combined impact of extremely high magnetic properties and (ii) possible formation of an oxidized Fe layer on the surface of nZVI particles [21,30]. The average particle size of nZVI-Bento was 13.4 nm (n = 14), as observed from the TEM micrographs (data not presented). The X-ray diffraction (XRD) analysis was conducted to assess the crystallinity and non-destructively perform a qualitative evaluation of the composition of ZVI products. The XRD pattern of both products revealed a distinct peak at 2θ = 44.9° (Figure S1a,b), which confirmed the characteristic peak of Fe^0^ [31,32]. In addition, the XRD analysis of nZVI-Bento also revealed two distinct peaks at 2θ = 31.4 and 5.8°, which corresponded to maghemite (γ-Fe_2_O_3_) [32] and Na-montmorillonite [33], respectively. Iron oxide formation may be attributed to the oxidation of Fe^0^ during the synthesis and drying processes.

#### 2.1.2. Chemical Properties

The elemental composition of the ZVI products was assessed via energy-dispersive X-ray fluorescence spectroscopy (ED-XRF). The nZVI-Bare in its pristine form (unmodified) contains 99.6% Fe (*w*/*w*) with traces of residual S and P that might have been left over from the sulphates salts of Fe during synthesis (Table S1). Conversely, after modification using bentonite clay, the Fe content dropped to 90.7% (*w*/*w*). Silicon (Si) was next with 5.37% (*w*/*w*), which was followed by Al (2.57%) and S (0.88%). The point of zero charge (ZPC) of the products was assessed by mass-titration method (Section S1). The ZPC of nZVI-Bare was higher, with a value of 9.0, while that of nZVI-Bento was 7.85 (Figure S3). This indicates that the unmodified ZVI needed a relatively higher pH to attain a net zero charge on its surface. The ZPC value of montmorillonite typically ranges between 2.5 and 3.0 [34,35]. The bentonite clay accoutred with Fe^0^ on the surface increased its ZPC value to a higher pH. The rise in ZPC value of nZVI-Bento (when compared to bentonite clay) may also be attributed to the oxides of Fe formed from Fe^0^ on the clay surface, which acquired more H^+^ ion on the surface, resulting in a shift in ZPC to a higher pH range.

### 2.2. Sorption Kinetics

Sorption kinetics study is required to comprehend the adsorbent–adsorbate interaction and decide the applicability of an adsorbent. The study describes the rate of adsorption or release of solutes from the solution to the adsorbent’s surface. The fitting of different kinetic models gives us a mechanistic insight of adsorption process. In this study, sorption kinetics was assessed, keeping the initial As(V) concentration in the solution to 5 mg L^−1^. This concentration was chosen because the maximum groundwater As concentration previously reported in India is 3.7 mg L^−1^ [7,8]. Conducting the batch kinetic study using the synthesized nano products revealed that both the products (nZVI-Bare/nZVI-Bento) efficiently removed >50% (~54% and ~52%, respectively) of As within the first 60 min. Later, As removal slowed down, which could be due to the saturation of adsorption surface and/or decrease in the As concentration in solution.

The rate of As removal plateaued at 24 h of the study with nZVI-Bare and nZVI-Bento removing ~99.6% and ~99.2% of the total As, respectively. Consequently, this time period was standardized for attaining equilibrium in all subsequent studies on removing As from water. The data were fitted to four kinetic models, i.e., pseudo-first order, pseudo-second order, Elovich, and intra-particle diffusion models. The slope and intercept from the linear equation was used to calculate the parameters presented in Table 1. Results reveal that the linearized forms of all four models fitted well with the data of both products. However, the pseudo-second-order equation had a relatively high R^2^ value of 0.99 for both products. This model could accurately predict the equilibrium adsorption capacity (*q_e_*) (20.5 and 21.0 mg g^−1^ for nZVI-Bare and nZVI-Bento, respectively), which was experimentally found to be 19.9 (nZVI-Bare) and 19.8 mg g^−1^ (nZVI-Bento), suggesting a chemisorption process [36]. This model is mainly related to the vacant active sites of adsorption, and deviates at higher solute concentrations [37,38]. The approaching equilibrium factor (*R_w_*) values were <<0.01 for both products, indicating a pseudo-rectangular kinetic curve that falls under zone III, meaning that the adsorption reaction reached equilibrium rapidly [39].

The data also fitted well with the pseudo-first order equation, with high R^2^ values of 0.83 and 0.91 for nZVI-Bare and nZVI-Bento, respectively. However, the model-predicted values of *q_e_* did not correspond well with the experimental values (Table 1). This model fitted well at high initial solute concentration (*C*_0_), thus causing a proportional decrease in the reaction rate constant (*k*_1_) [40]. Consequently, the value of the rate constant was significantly lower in the pseudo-first-order reaction (*k*_1_) compared to the pseudo-second-order (*k*_2_) in both products. This finding correlates with the adsorption mechanism, suggesting that physisorption is also involved in the As removal process by ZVI products; however, chemisorption dominates due to the high R^2^ value.

To understand the adsorption mechanism Elovich model was employed. This model is used to describe the chemisorption of liquid into solid and the heterogeneity of the adsorbent surface [41]. In this study, the Elovich model fitted well with the kinetic data, giving high R^2^ values of 0.96 and 0.97 for nZVI-Bare and nZVI-Bento, respectively. The presence of bentonite clay along with various Fe corrosion reaction products explains the heterogeneous surface of nZVI-Bento. This model gives an insight into the net retention of adsorbate by adsorbent based on the relative rate of adsorption and desorption. The value of the initial adsorption constant (*a*) was much higher than the desorption constant (*b*) in both products. This clearly reveals that the rate of adsorption of As by ZVI products exceeded the desorption rate, facilitating faster removal of As [42,43]. Both pseudo-first- and second-order models are widely used for characterizing the adsorption process; however, they do not explain the diffusion of adsorbate into adsorbent. For this, kinetic data were fitted to the intra-particle diffusion model. A linear curve representing *q_t_* vs. *t*^1/2^ was plotted for both ZVI products, and it was observed that in both cases, the line did not pass through (0, 0), indicating that adsorption of As by the adsorbents followed multiple mechanisms. In the case of ZVI modified by bentonite clay, apart from physi-and chemisorption, internal diffusion of As into the adsorbent also governed the faster removal of As (although this could be the secondary mechanism) from the solution.

### 2.3. Sorption Isotherm

Recently, the focus of researchers has been confined to devising innovative and cost-effective technologies to restrict the entry of pollutant elements into drinking/irrigation water for humans. While low-cost adsorbents have gained popularity, they require sorption characterization to understand the mechanisms and their adsorption potential [44]. In order to characterize the mechanism governing the As adsorption pathway in the case of nZVI-Bento, we fitted the sorption data into two widely used equilibrium sorption isotherm models, viz., Langmuir and Freundlich. The linearized equations of these models were employed for estimating the isotherm parameters (Table 2). The fitting of data to the Langmuir isotherm model (linear) showed that the adsorption maxima (*q_max_*) value of nZVI-Bare was 288 mg g^−1^, which was ~93% higher than that of nZVI-Bento (149 mg g^−1^). The *q_max_* of the newly synthesized product in the present study was very high compared to already published works on metal/metalloid removal by clay-supported ZVI products [18,21,45]. The lower *q_max_* values compared to nZVI-Bare could be attributed to the blocking of reactive sites of ZVI by bentonite clay in the form of a discrete clay cover. The Langmuir constant (*K_l_*) represents the binding energy or affinity of the sorbent for sorbate. Both products exhibited equal values of *K_l_* (0.017 L mg^−1^), indicating a similar affinity of As for the ZVI surface with or without bentonite. Moreover, the high R^2^ value (0.87 for nZVI-Bare and 0.84 for nZVI-Bento) indicated a good fit to the Langmuir model, which suggests the monolayer adsorption of As on the ZVI surface [21,46]. The value of *R_L_* in both nZVI-Bare (0.11–0.96) and nZVI-Bento (0.12–0.96) was <1, highlighting the favorable adsorption of As on the products’ surface [47]. The Freundlich model was the better fit for sorption data of As onto both ZVI products with R^2^ values of 0.98 (nZVI-Bare) and 0.92 (nZVI-Bento), which was higher than those of the Langmuir model (Table 2). The good fit of this model with the adsorption data reveals the energetic heterogeneity of the adsorption surface of the ZVI products [48]. The slope of the linear form of the Freundlich equation (1/*n*) explains the mechanism of adsorption to some extent. In this study, 1/*n* value was <1 for both products, confirming the chemisorption of As on the ZVI surface [49]. This also implies that adsorption of As can take place even at high As concentration [50].

The stability of ZVI products, as well as the feasibility of their repeated use, were assessed through desorption of pre-adsorbed As on ZVI. Results (data not shown) indicated that the three-step desorption of As in water from pre-adsorbed As from the ZVI product was almost non-detectable, showing a very high affinity of As for ZVI. The same solution was assessed for Fe release. Results showed that the cumulative release of Fe in three desorption steps (D1, D2, and D3) was 38.3 and 3.6 mg kg^−1^ in nZVI-Bare and nZVI-Bento, respectively (Figure S2). Such high stability of nZVI-Bento could be attributed to the protection imparted by bentonite clay to the ZVI core. After the sorption study, the ZVI products were filtered and dried for their XRD analysis. The characteristic peak of Fe^0^ was absent in the case of nZVI-Bare, with the emergence of some additional peaks at 2θ = 31.4 and 35.6° (corresponding to γ-Fe_2_O_3_ formation [32]) (Figure S1b). However, the characteristic peak of Fe^0^ (nZVI-Bento) remained intact even after the As adsorption study, indicating its higher stability and better shelf-life when compared to nZVI-Bare.

### 2.4. Non-Linear Regression Modeling of Sorption Isotherm and Kinetic Models

**Figure 2:**
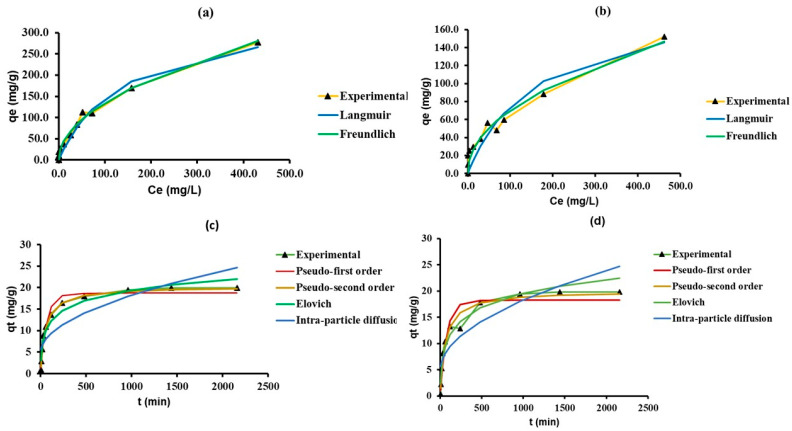
Comparison of experimental and model predicted adsorption capacity (mg g^−1^) values in various non-linear isotherm models; (**a**) nZVI-Bare, (**b**) nZVI-Bento and kinetic models, (**c**) nZVI-Bare, and (**d**) nZVI-Bento.

Linear data-fitting for deriving model parameters is an easy way to understand the mechanisms governing adsorption. One common way of modifying a non-linear model equation into linear form is to conduct a log transformation. However, this does not ensure constant variance among all adsorption data sets. Linear regression can glean homoscedastic data sets from the adsorption data sets with inconsistent variance values. However, in the process, this results in the inaccurate estimation of parameters endowed with high error values, thus making linear regression modelling more obscure. For this reason, in our study, we have fitted the non-linear form of both sorption isotherm and kinetic models, and compared the model parameters of linear and non-linear models which are presented in Table 1 and Table 2, respectively. The non-linear fitting was executed using OriginPro Lab 2021 software. The Levenberg–Marquardt algorithm served to conduct multiple iterations for solving the least square plot-fitting problem and minimizing the variance. The non-linear plots comparing the experimental and model predicted adsorption capacity values are presented in Figure 2.

The R^2^ values of all non-linear kinetic models were equal to (in the Elovich and intra-particle diffusion models) or higher (pseudo-first- and pseudo-second order models) than the linear kinetic models. The parameter values in Elovich and intra-particle diffusion models in both linear and non-linear regression fitting were comparable (Table 1). The non-linear pseudo-first order model predicted the value of *q_e_* to be around 18.7 and 18.3 mg g^−1^ for nZVI-Bare and nZVI-Bento, respectively, which was a much better approximation of experimental *q_e_* (19.9 (nZVI-Bare) and 19.8 mg g^−1^ (nZVI-Bento)) than linear regression. Referring to non-linear fitting of sorption isotherm models, the R^2^ value did improve in the Langmuir and Freundlich models (Table 2).

The adsorption maxima predicted by non-linear Langmuir model for nZVI-Bare and -Bento were 22.8% and 24.7% higher than the predicted values of linear regression. The plot between *q_e_* vs. *C_e_* showed an ‘L’-shaped adsorption isotherm curve, indicating strong adsorbate–adsorbent interaction at a low As concentration. This affinity diminished with the increase in solution As concentration, which can be attributed to the filling up of the active sites of adsorption in ZVI products.

### 2.5. Error Analysis for Testing Model Validation

Model suitability and finding out the best-fit model in both kinetics and sorption isotherm study is easily judged from the R^2^ value. This is further confirmed by the comparison of the closeness of model predicted and experimental *q_e_* values. However, it is not definite that the model fits perfectly with the data set. In fact, obtaining a high R^2^ value does not automatically mean the model is acceptable. Moreover, the belief that R^2^ ≥ 0.80 implies good model fit [51,52] contradicts the selection of a model with a R^2^ value closer to 1. Some additional mathematical error functions can be employed in order to validate a model’s suitability in further research for better prediction of experimental data and parameters therein.

**Figure 3:**
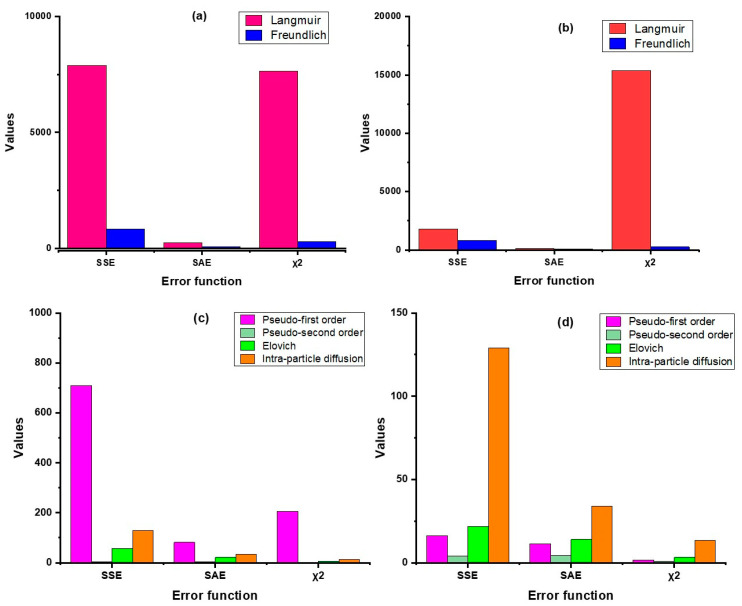
Error analysis of isotherm models (linear (**a**) and non-linear (**b**)) and kinetic models (linear (**c**) and non-linear (**d**)) for nZVI-Bare. SSE, error sum of squares; SAE, sum of absolute error; χ^2^, chi-square; AICc, corrected Akaike Information Criterion.

**Figure 4:**
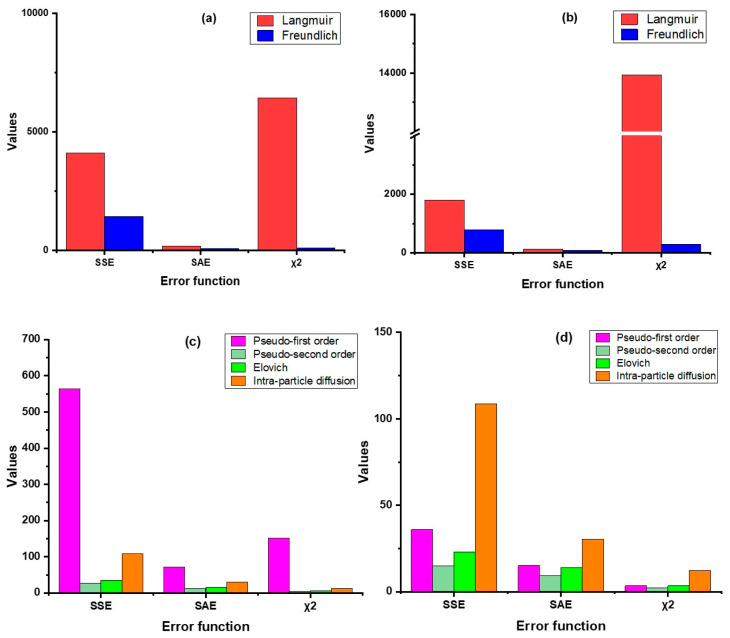
Error analysis of isotherm models (linear (**a**) and non-linear (**b**)) and kinetic models (linear (**c**) and non-linear (**d**)) for nZVI-Bento. SSE, error sum of squares; SAE, sum of absolute error; χ^2^, chi-square; AICc, corrected Akaike Information Criterion.

In this study, we have used four error functions viz. SSE, SAE, χ^2^, and AICc, of which AICc was used for testing goodness-of-fit (Figure 3 and Figure 4, Table S2). The results revealed that the non-linear regression models (both sorption kinetics and isotherm) had smaller error values compared to the linear models, except in the Langmuir model, where the value of χ^2^ was lower in linear regression as compared to non-linear. For both products, the pseudo-second-order model showed minimum error values in contrast to the other kinetic models. In the case of the adsorption isotherm models, the Freundlich model had the lowest error values. The AICc values substantiates this inference as the values of the pseudo-second order model (non-linear) were the lowest for both ZVI products, hovering around 57.5 (nZVI-Bare) and 71.9 (nZVI-Bento); the corresponding values for the Freundlich isotherm model were 105.5 and 105.1, respectively (Table S2). 

### 2.6. Factors Affecting As Removal

#### 2.6.1. Effect of pH

**Figure 5:**
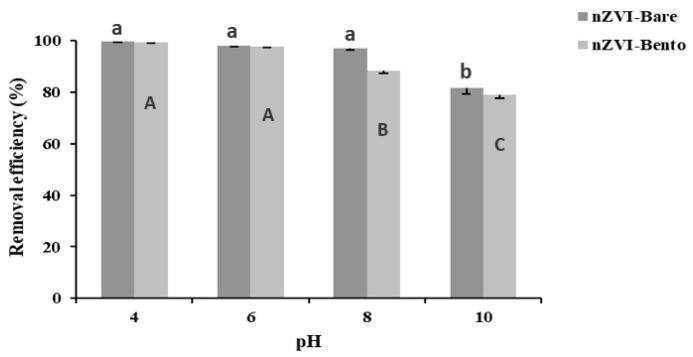
Effect of pH on arsenate (As (V)) removal (%) by nano ZVI products (adsorbent dose = 0.25 g L^−1^) from arsenic-contaminated water (*C*_0_ = 5 mg L^−1^). Different lowercase letters denote a significant difference among the values across the treatments in nZVI-Bare, while different uppercase letters denote significant difference for nZVI-Bento treatments.

A significant reduction in As removal efficiency was observed in nZVI-Bento (88.4%) at and above pH 8.0 (Figure 5). This could be attributed to the repulsion of arsenate (AsO_4_^3−^) ions on the negatively charged surface of nZVI-Bento, which was attained at pH > ZPC (7.85) [53,54]. However, for nZVI-Bare, the decrease in As removal was observed at pH = 10. This was due to higher ZPC value of nZVI-Bare (9.0) (Figure S3). This shows that the removal of As by nZVI products follows electrostatic force of attraction [29]. As much as 99.1% total As (*C*_0_ = 5 mg L^−1^) was removed at pH 4, which reduced to 97.6% at pH 6.0. Thus, removal of As by nZVI-Bento was pH-dependent and worked better within a pH range of 4–6.

#### 2.6.2. Effect of Adsorbent Dose

**Figure 6:**
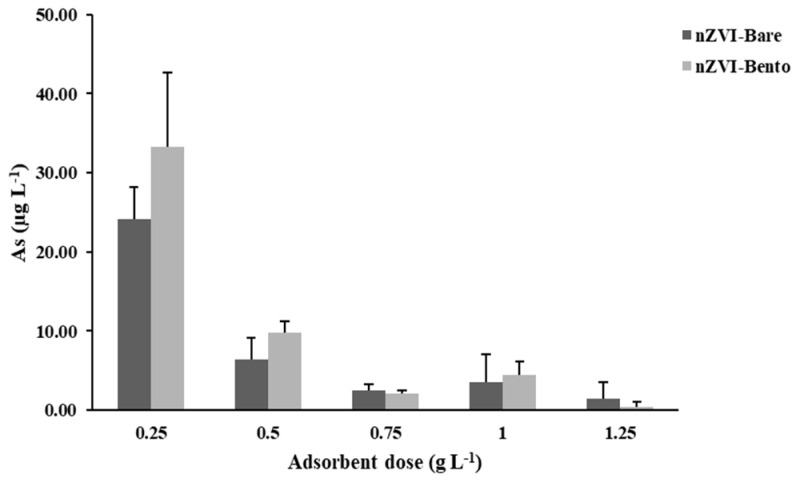
Effect of dose of ZVI products on As content of water with initial As (V) concentration of 5 mg L^−1^.

This study was carried out to optimize the dose of ZVI products for efficient removal of As from water to make it safe for human consumption and irrigation. The dose was standardized by considering the critical limit of As to be 10 µg L^−1^ [55]. An adsorbent dose of 0.5 g L^−1^ could successfully remove As (*C*_0_ = 5 mg L^−1^) in water to well below the safe limit of human consumption, which was much less than the critical limit of As for irrigation water (100 µg L^−1^) [55]. Thus, in effect, it was safe for both purposes (Figure 6).

#### 2.6.3. Effect of Ageing

The ZVI products are extremely reactive, thus contributing to quicker and efficient removal of pollutant elements from the medium. Such high reactivity is the reason behind its faster corrosion rate, affecting its efficiency. Ageing ZVI affects its reactivity by forming a layer of Fe corrosion products around the ZVI core. The Fe corrosion products include a mixture of Fe-based minerals such as amorphous Fe oxides and a series of Fe (hydr) oxides like maghemite, lepidocrocite, and magnetite [56,57]. Therefore, in this study, we have tried to evaluate the effect of ageing on the As removal efficiency of ZVI products (Section S2), which would give us an idea of its long-term storability without hampering its reactivity. The study shows that the efficiency of nZVI-Bare in removing As decreased significantly at only 15 days of ageing process, whereas for nZVI-Bento, the As removal efficiency remained consistent for up to 60 days and decreased significantly at 90 days of ageing (Figure S4). This could be attributed to the protection offered by bentonite clay covering the active sites of ZVI and preventing direct exposure of these sites to oxidation/reduction.

### 2.7. Application of ZVI Products for As Remediation in Soil

#### 2.7.1. Effect on Olsen-Extractable As Content in Soil

For soil application of ZVI products, As contaminated soils were collected from farmer fields, processed, and characterized for various physical and chemical properties, which are presented in Table 3.

**Figure 7:**
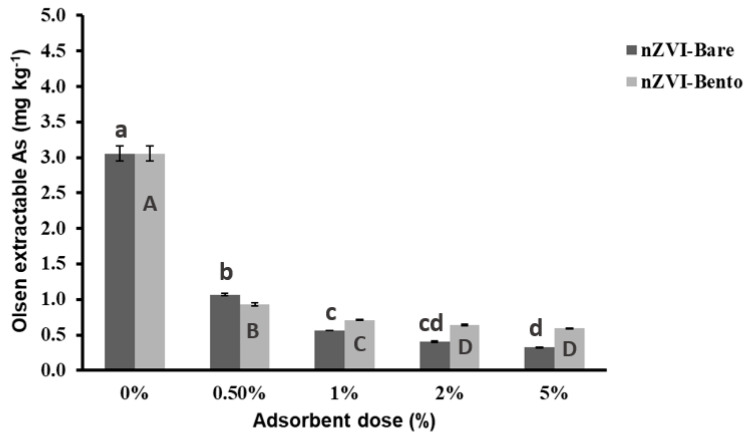
Effect of dose of ZVI products on Olsen extractable As content (mg kg^−1^) of As-contaminated soil collected from Mitrapur, West Bengal. Different lowercase letters denote a significant difference among the values across the treatments in nZVI-Bare, while different uppercase letters denote significant difference for nZVI-Bento treatments.

The soil was sandy clay in texture with alkaline pH (7.80) and had a high Walkley–Black organic carbon content of 20.4 g kg^−1^. The Olsen-extractable As content was 3.57 mg kg^−1^. The soil had a very high level of total As content of 32.2 mg kg^−1^ as compared to the world average of 10 mg kg^−1^ [26,55]. The soil was treated with graded concentration of ZVI products to optimize the dose of application for As remediation in soil. It was found that a significant reduction in Olsen-extractable As was achieved at 0.5% and 1% doses, bringing a reduction of 70.9% and 77.4%, respectively, for nZVI-Bento (Figure 7). However, with a subsequent increase in dose, there was negligible reduction in Olsen-extractable As levels in soil.

#### 2.7.2. Effect on As Fraction in Soil

Distribution of As in different inorganic As (i-As) fractions in soil as affected by dose (1% and 2%) ZVI products was studied; the data are presented in Table 4. Application of 1% and 2% dose of nZVI-Bento reduced the non-specifically sorbed As fraction in soil from 0.24 mg kg^−1^ (control) to 0.02 and 0.01 mg kg^−1^, respectively. This was further reduced to negligible quantities when similar doses of nZVI-Bare were applied to soil. Similar results were obtained by Kumpiene and co-workers [58], where application of 1% dose of unmodified nZVI resulted in the reduction of exchangeable As fraction from 6.7 mg kg^−1^ to untraceable levels. The specifically sorbed As fraction (F2) also decreased from 17% in control to as low as 4.6% and 3.7% at 1% and 2% doses of nZVI-Bare, respectively, while the corresponding values in nZVI-Bento were 4.7% and 3.6%, respectively (Figure S5). These findings corroborate the findings of Kim and co-workers [59], wherein As content in leachates of sequential extraction of nZVI-treated soil were significantly reduced in F1 and F2 fractions. 

Interestingly, the application of ZVI products had a remarkable effect on the stabilization of As bound to amorphous and crystalline hydrous oxides of Fe and Al fractions (F3). The application of 1% and 2% doses of nZVI-Bento resulted in an increase in the F3 fraction to as high as 45.3% and 64.3% higher than that of the control. It has been proposed in earlier works [60,61] that the ZVI transforms easily to hydrous oxides of Fe when applied to soil. This further exposes a fertile surface for more adsorption of As, facilitating higher As removal and its stabilization in soil. This explains the noteworthy increase in F3 fraction after treatment of soil with ZVI products. These results were consistent with that of nZVI-Bare treated soils. The layer of Fe corrosion products on the surface of ZVI forms ligand complexation of Fe–As compounds, which are very stable. Due to the continuous ageing of ZVI in soil leading to freshly formed Fe oxide/hydrous oxide mineral formation on the surface, the ferro-arsenic complexation reaction continues in the forward reaction. This causes a significant reduction in the water-soluble and exchangeable fraction in soil with a corresponding increase in the amorphous and crystalline hydrous oxides of Fe and Al fraction. The fraction of As bound to well-crystallized hydrous oxides of Fe, and Al decreased significantly at a 2% dose of nZVI-Bento, resulting in a 25.4% reduction over the control. It is evident that the F1 and F2 fractions of As determined in the sequential fractionation scheme of Wenzel and co-workers [62] correlated with plant uptake of As [63,64], indicating a major contribution of these fractions to plant As content. Therefore, the significant reduction in As in F1 + F2 fractions reduces the risk of phytoavailability of As to plants, indicating a low transfer of As to the human food chain.

## 3. Materials and Methods

### 3.1. Chemicals

Sodium arsenate (Na_2_HAsO_4_·7H_2_O) (AR (ACS) grade) was procured from the Central Drug House (CDH) (India). Sodium borohydride (NaBH_4_) (EMPLURA^®^), iron (II) sulphate heptahydrate (FeSO_4_·7H_2_O) (EMPLURA^®^), and sodium hydroxide (NaOH) (EMPARTA^®^) were purchased from Merck SA (Darmstadt, Germany). Nitrogen (N_2_) gas (grade-1) was procured from Sigma Gas Services, New Delhi, India. In all experiments, ultrapure Milli-Q water was used, which was prepared using a Milli-Q^®^ direct water purification system purchased from Merck SA (Germany). For preparing standard As(V) solutions, 1000 mg L^−1^ As inductively coupled plasma (ICP) standard stock solution was purchased from Supelco^®^ Sigma Aldrich, Merck (Darmstadt, Germany). The commercial grade bentonite clay was procured from SD Fine-Chem Limited (SDFCL) (Mumbai, India).

### 3.2. Synthesis of Bentonite-Supported Nano Zero-Valent Iron

For synthesis of the engineered nZVI product, the protocol proposed by Das and co-workers [46] was followed with some modifications. These changes were required to minimize the production cost of the new nZVI product and ensure its economic feasibility. A mass of 0.25 g of commercial grade bentonite clay was added to 100 mL of Milli-Q water in a three-necked flask and placed over a hot plate/magnetic stirrer equipped with a N_2_ gas line. The suspension was stirred with a magnetic stirrer at 400 rpm for 30 min while continuously purging N_2_ gas into the mixture to deoxygenate the bentonite solution. Thereafter, 3 g of ferrous sulphate heptahydrate (FeSO_4_·7H_2_O) was added to the reaction flask, followed by the slow addition of 50 mL Milli-Q water upon continuous stirring for the next 30 min. Then, the pH of the solution was adjusted to 6.0 by using 1 M NaOH solution. By raising the pH of the solution, oxidation of Fe^2+^ occurred, forming particles of ferrihydride that precipitated and acted as nuclei for forming precipitates of nZVI [65]. The temperature was raised to 60 °C on the hot plate/magnetic stirrer. Then, sodium borohydride (NaBH_4_) (reducing agent) solution (1 g in 50 mL Milli-Q water) was added drop-wise, and the mixture was continuously stirred with N_2_ gas addition for the next 4 h until the reaction reached completion.

The solution containing black-colored precipitate of bentonite-supported nano zero-valent iron (nZVI-Bento) was transferred to 50 mL centrifuge tubes, then centrifuged at 5000 rpm. The supernatant was decanted and stored in a bottle for further analysis. The precipitates formed in the 50 mL tubes were washed twice each using Milli-Q water and ethyl alcohol, followed by final washing using Milli-Q water to ensure removal of any excess NaBH_4_. The amount of NaBH_4_ added was one third of the amount of FeSO_4_·7H_2_O added (NaBH_4_: FeSO_4_·7H_2_O::1:3). This was standardized such that no ionic form of Fe^2+/3+^ remained in the supernatant, which was ensured by colorimetric estimation of Fe using the O-phenanthroline method [66]. The black solids of nZVI present in the centrifuge tubes were dried in a vacuum desiccator to avoid corrosion of the ZVI product. After drying, the nZVI-Bento was ground using a porcelain mortar and pestle and subsequently stored in a polypropylene box. For synthesis of unmodified nZVI (nZVI-Bare), the same protocol was followed without adding bentonite clay. The chemical reaction for synthesis of ZVI is presented below.

Fe^2+^ (aq) + BH_4_^−^ → Fe^0^ ↓ + B(OH)_3_ + H_2_ (g)

### 3.3. Characterization of nZVI Products

The X-ray diffraction analysis was conducted using a Philips PW 1710 X-ray diffractometer with APD (automated powder diffraction) software and the following instrumental set-up: radiation type, Cu-Kα (λ = 1.5418 Å); generator voltage, 40 kV; tube current, 20 mA; angle (2θ) range, 4°–50°. Surface characterization of nano products was conducted using a scanning electron microscope (SEM) (Zeiss EVO series, EVO 50, resolution of 2.0 nm at 30 kV). The microstructure and particle size of the synthesized products were examined using transmission electron microscopy (TEM) (JEOL TEM model JEM-1101 (JEOL Ltd., Tokyo, Japan)). The elemental compositions of powdered samples were assessed with an Epsilon 4 50Kv (Malvern Panalytical, The Netherlands) energy dispersive X-ray fluorescence spectroscopy (ED-XRF) equipped with a high-resolution Si drift detector. 

Analysis of As was conducted using ICP mass spectroscopy (PerkinElmer NexION 300). For the incubation study, REMI orbital shaking incubators were employed. Meanwhile, the point of zero charge (ZPC) was measured following the mass titration method [67] (Section S1).

### 3.4. Sorption of As by nZVI Products

A batch sorption study for arsenate (As(V)) sorption was conducted with nZVI-Bento and nZVI-Bare serving as the sorbent. For this purpose, 7.5 mg of ZVI product (0.25 g L^−1^) was taken in centrifuge tubes. A volume of 30 mL of solution containing graded concentrations of 0, 2.5, 5, 10, 20, 40, 60, 80, 100, 200, and 500 mg As L^−1^ was added to these tubes and shaken at 120 rpm in an orbital shaking incubator for 24 h at 25 °C. The content of the tubes were centrifuged at 5000 rpm for 10 min and filtered out using Whatman 42 filter paper. The As content in the filtrate was determined using an ICP-MS.

Adsorbed As (*q_e_*) was computed as follows:

(1) $$q_{e}=\frac{{(C}_{0}-C_{e})V}{w}$$

where *C*_0_ and *C_e_* are the initial As concentration and equilibrium As concentration, respectively; *V* is volume of solution taken; and w denotes weight of adsorbent taken.

Sorption isotherm: sorption data were summarized with the help of Langmuir and Freundlich sorption isotherms. The Langmuir model is semi-mechanistic in nature, whereas the Freundlich model is purely empirical. The Langmuir model is expressed as

$$q_{e}=\frac{q_{maxC_{e}K_{l}}}{1+K_{l}C_{e}};$$

which is linearized to

(2) $$\frac{C_{e}}{q_{e}}=\frac{C_{e}}{q_{m}}+\frac{1}{q_{mK_{l}}}$$

The sorption intensity (*R_L_*) represented a dimensionless constant [44], which is calculated using the following equation:

(3) $$R_{L}=\frac{1}{1+C_{0}K_{l}}$$

This value indicates favorability of the adsorption process by the adsorbent. If it is unfavorable, then *R_L_* > 1; it is favorable if 0 < *R_L_* < 1; and it is irreversible if *R_L_* = 0.

The Freundlich isotherm model is expressed as

$$q_{e}=K_{f}{C_{e}}^{1/n}$$

which is linearized to

(4) $$\log q_{e}=\log K_{f}+\frac{1}{n}\log C_{e}$$

where *q_max_* (mg g^−1^) represents the maximum adsorption capacity, *K_l_* (L mg^−1^) stands for the Langmuir constant (representing affinity between the solute and adsorbent), *K_f_* is the Freundlich constant, and 1/*n* denotes the adsorption intensity (1 < *n* < 10).

### 3.5. Sorption Kinetic Study

For the kinetic studies, an initial As concentration of *C*_0_ = 5 mg As L^−1^ was used. An adsorbent (nZVI-Bento and nZVI-Bare) dose of 0.25 g L^−1^, i.e., 7.5 mg ZVI, was placed in centrifuge tubes containing 30 mL of As solution and shaken in an environmental shaker at 25 °C for different time intervals, viz., 5, 10, 20, 30, 60, 120, 240, 480, 960, 1440, and 2160 min. The tubes were withdrawn at these time intervals, and the contents were filtered after centrifugation at 5000 rpm for 10 min. The As concentrations in these solutions were measured using ICP-MS. The data were used to determine the reaction order. 

Amount of As adsorbed in time *t* (*q_t_*) was calculated by the following equation:

(5) $$q_{t}=\frac{{(C}_{0}-C_{t})V}{w}$$

where *C_t_* is the equilibrium As concentration in the filtrate at time *t*.

#### 3.5.1. Pseudo-First Order Kinetic Model

A typical example of the two parameter model is Lagergren’s equation [68]. This is particularly accurate when the initial solution concentration is high. They are actually higher order reactions, but appear to behave as first-order ones, thus obtaining the name pseudo-first order reaction. The reaction rate depends on two reactants, but it appears as if it is decided by the concentration of only one.

(6) $$A+B\overset{k_{1}}{\to }P$$

If ‘*A*’ and ‘*B*’ are reacting to deliver ‘*P*’ product with a rate constant of *k*_1_ (min^−1^), then the above reaction strongly suggests a second-order reaction, since the rate *k*_1_ depends on the concentrations of *A* and *B*. If the concentration of [*B*] ˃˃ [*A*], then any change in [*B*] is negligible with time. Thus, *k*_1_ depends on the change in [*A*], and the reaction behaves as a first-order reaction, even though it is of a higher order.

The equation is presented as

(7) $$q_{t}=q_{e}(1-e^{-k_{1}t})$$

while the linearized form of the equation is

(8) $$\ln\left(q_{e}-q_{t}\right)=lnq_{e}-k_{1}t$$

#### 3.5.2. Pseudo-Second Order Kinetic Model

This is another popular kinetic model for characterizing the adsorption kinetic process. It was originally first applied to study the adsorption of lead on peat by Ho and McKay [69]. This model confirms the occurrence of a chemical sorption process between adsorbent and adsorbate. Unlike the pseudo-first order reaction, the pseudo-second order originally has third-order kinetics, which are approximated as second-order due to the rate-limiting step of chemisorption. The equation is written as follows:

(9) $$q_{t}=\frac{{q^{2}}_{e}k_{2}t}{1+q_{t}k_{2}t}$$

The linearized form of the equation is presented as follows:

(10) $$\frac{t}{q_{t}}=\frac{1}{k_{2}q_{e}^{2}}+\frac{t}{q_{e}}$$

where *k*_2_ is the pseudo-second order rate constant (min^−1^).

The kinetics of the curve are characterized by employing an approaching equilibrium factor (*R_w_*), which is estimated using the following equation:

(11) $$R_{w}=\frac{1}{1+q_{e}k_{2}t_{ref}}$$

Here, *t_ref_* is the highest time recorded in the kinetics experiment.

#### 3.5.3. Elovich Model

This model was originally devised for the chemical adsorption of gas into solid. However, quite recently, it has been extrapolated to understand the mechanism of sorption of liquid into solids. It assumes that the sorbent surface is heterogeneous and that, with time, there is an increase in the activation energy. The model describes an exponential decline in the adsorption of adsorbate when the concentration of sorbent increases [70,71].

The non-linear form of this model is given as follows:

(12) $$\frac{dq}{dt}=ae^{-bq_{t}}$$

This equation is linearized in the form given below.

(13) $$qt=\frac{1}{b}\ln\left(ab\right)+\frac{1}{b}ln(t)$$

Here, *a* (mg g^−1^ min^−1^) and *b* (g mg^−1^) are initial adsorption rates and the desorption constant, respectively.

#### 3.5.4. Intra-Particle Diffusion Model

Diffusion models have been proposed to be of two types, i.e., external and internal, based on the rate-limiting step. The model presented decades ago by Weber and Morris [72] is a type of internal diffusion model indicating instantaneous diffusion of sorbate into and around the sorbents’ active sites. This model is widely employed and presented as:

(14) $$q_{t}=k_{int}t^{1/2}$$

The linearized form of the above equation is:

(15) $$q_{t}=k_{int}t^{1/2}+C_{int}$$

where *k_int_* (mg g^−1^ min^−1/2^) is the rate constant for intra-particle diffusion, and *C_int_* (mg g^−1^) is the constant related to boundary layer thickness.

### 3.6. Effect of pH on Arsenic Removal

The pH of the solution with an initial As(V) concentration of 5 mg L^−1^ was adjusted to 4, 6, 8, and 10 using 0.1 N HCl or NaOH. Using a fixed adsorbent dose of 0.25 g L^−1^, the adsorption study was carried out in centrifuge tubes for 24 h, as was done for the sorption isotherm analysis. Then, the solution was centrifuged and filtered for As estimation using an ICP-MS. The arsenic removal efficiency was calculated using the following equation:

(16) $$Removal\;efficiency\;(\%)=\frac{C_{0}-C_{e}}{C_{0}}\times 100$$

### 3.7. Optimization of Adsorbent Dose for Arsenic Removal

This study was executed to standardize the dose of sorbents (ZVI products) for remediation of As-contaminated water so that it was fit for drinking and irrigation. For this purpose, graded doses of sorbents were taken in the range of 0.25 to 1.25 g L^−1^ in 30 mL of solution (initial As concentration (*C*_0_) = 5 mg L^−1^) and shaken in an orbital shaking incubator for 24 h at 25 °C. Then, after centrifugation and filtering out the supernatant, the As content was measured using ICP-MS, and As removal efficiency (%) was calculated.

### 3.8. Soil Sampling and Characterization

Arsenic contaminated bulk soil samples (0–15 cm) were collected from farmer fields in Mitrapur region, Nadia district, West Bengal, India. The samples were air-dried, ground, and passed through a 2 mm sieve. The processed soil sample was characterized for various physical and chemical properties. The pH and electrical conductivity (EC) were measured in a soil:water (1:2) suspension and supernatant, respectively, utilizing a combined digital pH and conductivity meter (Eutech PC 700, Thermo Fisher Scientific Inc., India) [73]. The hydrometer method was used to determine the mechanical composition of soil [74], and the soil’s texture was found using the texture triangle proposed by USDA [75]. The organic carbon content in soil was determined by the wet oxidation technique [76], while the cation exchange capacity (CEC) of soil was measured by ammonium acetate method [77]. Aqua Regia [78] and Olsen-extractable [79] As content in soil were measured by ICP-MS.

### 3.9. Effect of ZVI Dose on Extractable As and As Fractions in Soil

The processed soil sample (50 g) was put into a polypropylene box, and ZVI products were added at 0.5, 1, 2, and 5% (*w*/*w*). Water was added to soil up to 70% of water holding capacity, and the box was incubated for 30 days in an orbital shaking incubator at 25 °C. After incubation, soil was air-dried and processed for analyzing Olsen-extractable As content [79]. Distribution of As in different fractions was determined in soils treated with ZVI at 1 and 2% (*w*/*w*), following the sequential fractionation protocol [62].

### 3.10. Error Analysis and Data Presentation

To identify the best-fit model, functional errors were examined. In this study, the experimental (actual) and predicted data (from various models) were compared, and the best-fit model was identified based on various error functions, namely error sum of square (SSE), sum of absolute error (SAE), and chi-square (χ^2^). For goodness-of-fit, the corrected Akaike Information Criterion (AICc) was calculated, and the model with the lowest AICc was selected. 

These functions were estimated using the following equations:

(17) $$SSE=\sum_{i}^{N}{{(q}_{exp}-q_{pred})}_{i}^{2}$$

(18) $$SAE=\sum_{i}^{N}{\left|q_{exp}-\left.q_{pred}\right|\right.}_{i}$$

(19) $$\chi^{2}=\sum_{i}^{N}{\left[\frac{{\left(q_{exp}-q_{pred}\right)}^{2}}{q_{pred}}\right]}_{i}$$

(20) $$AICc=N\cdot \ln\left(2\pi SSE\right)+\frac{N\cdot (1+\frac{p}{N})}{1-(p+2)/N}$$

In the above equations, *q_exp_* and *q_pred_* are experimental and model predicted adsorption capacity (mg g^−1^), respectively. *N* is the total number of data points taken in the models, and *p* is the number of parameters in the model. All the data presented are mean values along with the standard deviation unless otherwise specifically mentioned.

## 4. Conclusions

The present study was formulated to address current needs for a low-cost and efficient sorbent product for the remediation of As from contaminated soils and water. An engineered-bentonite clay-based nano zero-valent iron material was synthesized, and it exhibited super-adsorbent features for removing As from soil and water at a very low dose of application. The novel product (nZVI-Bento @ 0.5 g L^−1^) could be successfully employed to reduce the As content of contaminated water (As concentration = 5 mg L^−1^). The mechanisms governing such high adsorption capacity of nZVI-Bento were also studied. It was deduced that a combined effect of chemisorption, physisorption, internal diffusion, and complexation/precipitation reactions could be the reason behind such higher efficacy of ZVI products. While the unmodified ZVI had higher As sorption capacity compared to nZVI-Bento, the latter was found to be much more resistant to corrosion. This property of nZVI-Bento makes this product particularly applicable for removing As from water over a longer period of time. We expect that the protocol for synthesizing this novel nZVI-Bento product could be scaling up for industry. 

The product was also successfully employed for the remediation of As-contaminated soil. A 1% (*w*/*w*) dose of nZVI-Bento could significantly reduce Olsen-extractable As content in As-contaminated soil while concomitantly stabilizing the soil As fractions by reducing the specifically and non-specifically bound As fractions (F1 + F2) and increasing the F3 fraction (As bound to amorphous and crystalline hydrous oxides of Fe and Al). This study may be able to offer guidance when devising future super-adsorbent nano-products for pollutant element remediation in soil and water or during their implementation. However, field-level studies assessing the efficacy of the novel product and the long-term effect of nano-products on soil ecological functions needs to be evaluated to allow widespread application.

## Figures and Tables

**Table 1:** Kinetic model parameters of As (V) sorption on ZVI products.

Kinetic Model	Kinetic Parameters	Linear		Non-Linear *	
		nZVI-Bare	nZVI-Bento	nZVI-Bare	nZVI-Bento
Pseudo-first-order	*q_e exp_* (mg g^−1^)	19.9	19.8	19.9	19.8
	*q_e_* (mg g^−1^)	10.4 ± 1.3	10.9 ± 0.69	18.7 ± 0.59	18.3 ± 0.89
	*K*_1_ (min^−1^)	0.004 ± 0.001	0.004 ± 0.0004	0.012 ± 0.002	0.013 ± 0.003
	R^2^	0.83	0.91	0.97	0.94
Pseudo-second-order	*q_e exp_* (mg g^−1^)	19.9	19.8	19.9	19.8
	*q_e_* (mg g^−1^)	20.5 ± 0.09	21.0 ± 0.42	20.3 ± 0.33	20.0 ± 0.72
	*K*_2_ (min^−1^)	157.5 ± 17.7	95.2 ± 21.5	0.001 ± 0.0001	0.0001 ± 0.0002
	R^2^	0.99	0.99	0.99	0.97
	*R_w_*	1.4 × 10^−7^	2.3 × 10^−7^	0.02	0.18
Elovich model	*a* (mg g^−1^ min^−1^)	2.68 ± 0.34	1.72 ± 0.11	1.00 ± 0.32	0.65 ± 0.18
	*b* (g mg^−1^)	0.32 ± 0.003	0.32 ± 0.003	0.29 ± 0.03	0.26 ± 0.03
	R^2^	0.96	0.97	0.96	0.97
Intra-particle diffusion model	*K_int_* (mg g^−1^ min^−0.5^)	0.43 ± 0.005	0.44 ± 0.008	0.43 ± 0.08	0.44 ± 0.07
	*C_int_* (mg g^−1^)	4.69 ± 0.18	4.18 ± 0.48	4.69 ± 1.63	3.91 ± 1.49
	R^2^	0.76	0.80	0.76	0.80

* Data represent mean ± SE.

**Table 2:** Sorption model parameters for As (V) sorption on ZVI products.

Sorption Model	Adsorption Parameter	Linear		Non-Linear *	
		nZVI-Bare	nZVI-Bento	nZVI-Bare	nZVI-Bento
Langmuir	*q_max_* (mg g^−1^)	288.5 ± 47.9	149.3 ± 61.2	354.3 ± 30.8	198.5 ± 34.4
	*K_l_* (L mg^−1^)	0.017 ± 0.003	0.017 ± 0.008	0.007 ± 0.001	0.006 ± 0.002
	R^2^	0.87	0.84	0.97	0.90
Freundlich	1/*n*	0.51 ± 0.06	0.35 ± 0.08	0.50 ± 0.03	0.48 ± 0.05
	*K_f_* (L g^−1^)	13.1 ± 3.49	13.6 ± 4.9	13.5 ± 1.93	7.71 ± 2.03
	R^2^	0.98	0.92	0.98	0.94

* Data represent mean ± SE.

**Table 3:** Characterization of arsenic-contaminated soil collected from farmer fields in Mitrapur, West Bengal, India.

Characteristics	Values
pH	7.80
Electrical conductivity (dS m^−1^)	0.88
Texture	Sandy clay
Sand %	43.6
Silt %	8.50
Clay %	47.9
Walkley–Black organic carbon (g kg^−1^)	20.4
Cation exchange capacity (cmol (+) kg^−1^)	27.8
Olsen-extractable As (mg kg^−1^)	3.10
Total As (mg kg^−1^)	32.2

**Table 4:** Effect of application of graded doses of ZVI product on distribution of As in different fractions in As-contaminated soil.

Treatment	Dose	Arsenic Content (mg kg^−1^) (Mean ± SD) in Different Fractions in Soil						Recovery (%)
		F1	F2	F3	F4	F5	Total	
Control	-	0.24 ± 0.02	5.66 ± 0.29	9.98 ± 0.72	7.54 ± 0.27	9.84 ± 1.15	33.3 ± 2.21	103.3
nZVI-Bento	1%	0.018	1.50 ± 0.09	14.5 ± 0.43	7.45 ± 0.11	9.44 ± 0.44	32.9 ± 0.80	102.1
	2%	0.008	1.25 ± 0.03	16.4 ± 0.51	8.66 ± 0.27	7.34 ± 0.33	33.7 ± 0.63	104.7
	LSD (*p* ≤ 0.05)	0.02	0.35	1.16	0.47	1.48	NS	
nZVI-Bare	1%	0.004	1.58 ± 0.11	15.4 ± 0.52	8.06 ± 8.06	8.29 ± 0.20	33.3 ± 0.90	103.6
	2%	0.001	1.26 ± 0.08	17.3 ± 0.39	9.13 ± 0.14	6.85 ± 0.31	34.6 ± 0.14	107.3
	LSD (*p* ≤ 0.05)	0.02	0.37	1.20	0.65	1.42	NS	

F1, non-specifically sorbed; F2, specifically sorbed; F3, amorphous and crystalline hydrousoxides of Fe and Al; F4, well-crystallized hydrous oxides of Fe and Al; F5 residual; LSD (*p* ≤ 0.05), least significant difference at 5% level of significance; SD, standard deviation.

## Data Availability

Not applicable.

## Supplementary Materials

The following supporting information can be downloaded at https://www.mdpi.com/article/10.3390/molecules28052168/s1. Figure S1: X-ray diffraction graph of ZVI products before and after sorption (AS) study for (a) nZVI-Bento and (b) nZVI-Bare; Figure S2: Release of iron (Fe) (mg L^−1^) from ZVI products in three subsequent desorption steps (D1, D2 and D3); Figure S3: Graph showing plot of experimental data of pHeq vs. adsorbent dose (g L^−1^) of ZVI products; Figure S4: Effect of ageing of ZVI products on As removal efficiency (%) of arsenate (As (V)); Figure S5: Effect of treatment of ZVI products and its dose of application on changes in As content in different fractions of As in soil. F1- Non-specifically sorbed; F2- specifically sorbed; F3- amorphous and crystalline hydrousoxides of Fe and Al; F4- well crystallised hydrousoxides of Fe and Al; F5- residual; Table S1: Elemental composition (% w/w) of ZVI products as measured using ED-XRF; Table S2: Corrected Akaike Information Criterion (AICc) values for different isotherm and kinetic models; Section S1: Assessment of point of zero charge (ZPC) of ZVI products; Section S2: Effect of ageing of sorbents on arsenic removal. Reference [67] is cited in the supplementary material.
