# Supplementary material for: Synthesis and Performance Evaluation of Novel Bentonite-Supported Nanoscale Zero Valent Iron for Remediation of Arsenic Contaminated Water and Soil
Source: Molecules. 2023 Feb 25;28(5):2168. doi: 10.3390/molecules28052168 (PMC10004430; doi:10.3390/molecules28052168)
Supplement: Supplementary file 1 [file molecules-28-02168-s001.zip › molecules-2238316-supplementary.pdf]

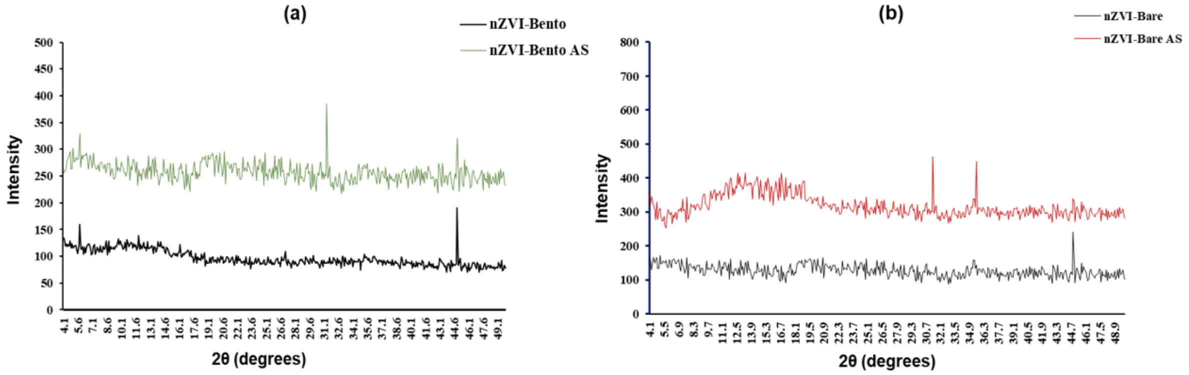

Figure S1. X-ray diffraction graph of ZVI products before and after sorption (AS) study for (a) nZVI-Bento and (b) nZVI-Bare.

Table S1. Elemental composition (% w/w) of ZVI products as measured using ED-XRF

Element (%)	nZVI- Bare	nZVI- Bento
Fe	99.6	90.7
Al	-	2.57
Si	-	5.37
S	0.27	0.88
P	0.13	0.15
K	-	0.23
Mg	-	0.10

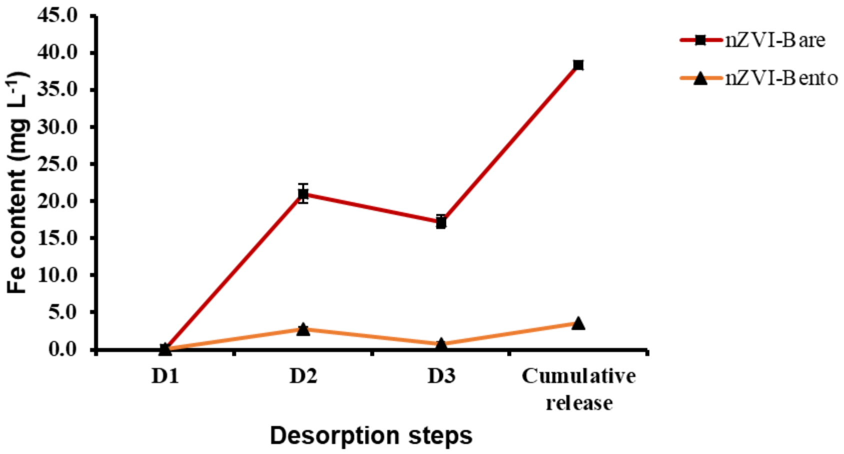

Figure S2. Release of iron (Fe) (mg L^{-1}) from ZVI products in three subsequent desorption steps (D1, D2 and D3).

Table S2. Corrected Akaike Information Criterion (AICc) values for different isotherm and kinetic models

Models	Linear		Non-linear	
	nZVI- Bare	nZVI- Bento	nZVI- Bare	nZVI- Bento
		Kinetic models		
Pseudo-first order	114.4	111.9	73.0	81.6
Pseudo-second order	56.9	78.2	57.5	71.9
Elovich model	86.9	81.5	76.2	76.7
Intra-particle diffusion model	95.7	93.8	95.7	93.8
		Isotherm models		
Langmuir	128.1	121.6	113.4	113.3
Freundlich	105.7	111.1	105.5	105.1

Section S1. *Assessment of point of zero charge (ZPC) of ZVI products*

The point of zero charge (ZPC) was measured following the mass titration method [67]. The ZVI products were suspended in 0.1 *M* $NaNO_3$ solution at doses ranging from 0.25 g L^{-1} to 50 g L^{-1}. After equilibration in an orbital shaking incubator at 20ºC for 24 h, the pH of the solution was measured. The ZPC was calculated from the pH vs mass curve at the point where the plot flatlined.

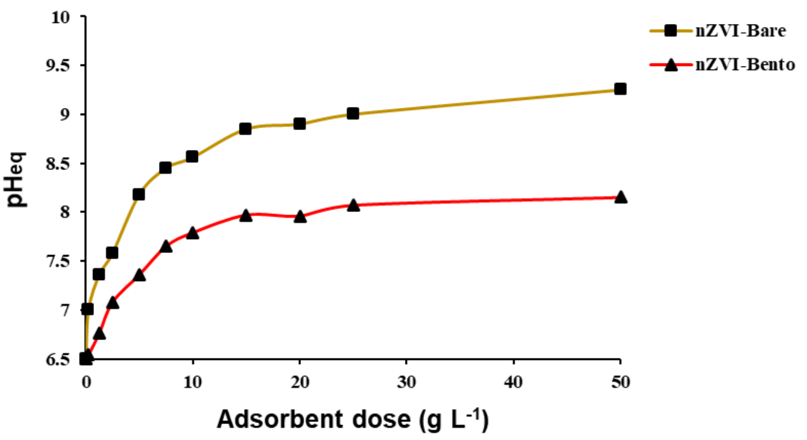

Figure S3. Graph showing plot of experimental data of pH_{eq} vs adsorbent dose (g L^{-1}) of ZVI products.

Section S2. *Effect of ageing of sorbents on arsenic removal*

The ZVI products were incubated in an open petri dish at 25 ºC for 90 days. The product was sampled periodically at 0, 15, 30, 60 and 90 days of incubation. These were used further for studying the As removal efficiency (sorbent dose = 0.25 g L^{-1}, C_0 = 5 mg L^{-1}).

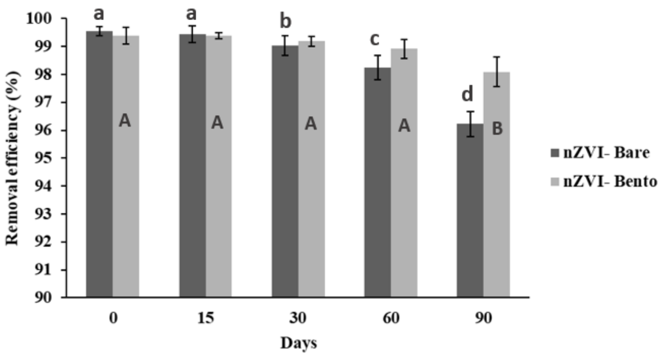

Figure S4. Effect of ageing of ZVI products on As removal efficiency (%) of arsenate (As (V)).

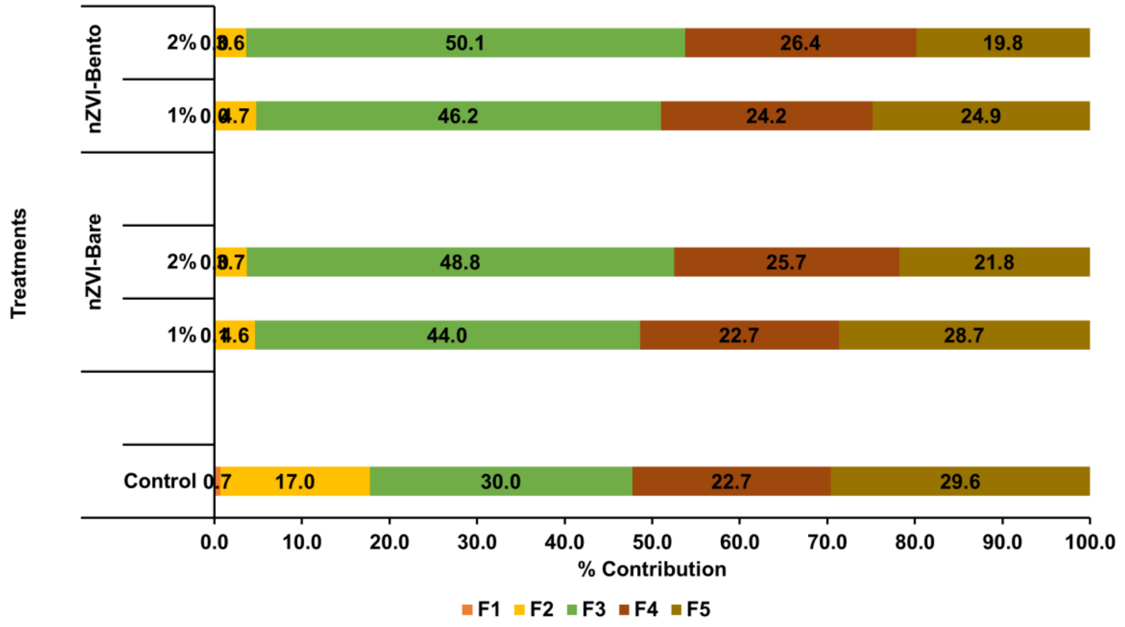

Figure S5. Effect of treatment of ZVI products and its dose of application on changes in As content in different fractions of As in soil. F1- Non-specifically sorbed; F2- specifically sorbed; F3- amorphous and crystalline hydrousoxides of Fe and Al; F4- well crystallised hydrousoxides of Fe and Al; F5- residual
